# ADT‐OH synergistically enhanced the antitumor activity of celecoxib in human colorectal cancer cells

**DOI:** 10.1002/cam4.6342

**Published:** 2023-07-26

**Authors:** Huangru Xu, Ping Li, Hailin Ma, Yuanhao Tan, Xiaoyang Wang, Fangfang Cai, Jiaqi Xu, Huisong Sun, Hongqin Zhuang, Zi‐Chun Hua

**Affiliations:** ^1^ The State Key Laboratory of Pharmaceutical Biotechnology, College of Life Sciences Nanjing University Nanjing P.R. China; ^2^ School of Biopharmacy China Pharmaceutical University Nanjing China; ^3^ Changzhou High‐Tech Research Institute of Nanjing University and Jiangsu TargetPharma Laboratories Inc. Changzhou P.R. China

**Keywords:** ADT‐OH, celecoxib, colorectal cancer, drug combination strategy

## Abstract

**Background:**

Colorectal cancer is one of the most prevalent cancers in the world, but the research on its prevention, early diagnosis and treatment is still a major challenge in clinical oncology. Thus, there is a pressing requirement to find effective strategies to improve the survival of colon cancer patients.

**Methods:**

Celecoxib has been accounted to be an effective antitumor drug, but may exhibit significant side effects. In recent studies, 5‐(4‐hydroxyphenyl)‐3H‐1,2‐dithiole‐3‐thione (ADT‐OH), one of the most commonly used reagents for the synthesis of sustained‐release H_2_S donors, has also been reported to inhibit cancer progression by affecting processes such as cell cycle, angiogenesis, and apoptosis. Therefore, we evaluated the therapeutic effect of the combination of ADT‐OH and celecoxib on colorectal cancer through in vitro and in vivo, hoping to achieve better therapeutic effect and reduce the effect of celecoxib on gastric injury through exogenous administration of H_2_S.

**Results:**

Our results demonstrated that ADT‐OH combined with celecoxib synergistically inhibited the proliferation and migration ability of human colorectal cancer HCT116 cells, altered cell cycle and cytoskeleton, increased intracellular reactive oxygen species (ROS), and promoted cell apoptosis. Noteworthy, in vivo studies also indicated the excellent antitumor therapeutic effect of the combination therapy without apparent toxicity.

**Conclusions:**

In general, our results provide a reasonable combination strategy of low‐dose ADT‐OH and celecoxib in the preclinical application of colorectal cancer.

## Graphical abstract

ADT‐OH combined with celecoxib synergistically inhibited the proliferation and migration ability of human colorectal cancer HCT116 cells, altered the cell cycle and cytoskeleton, increased intracellular ROS, and promoted cell apoptosis (Figure 8). Furthermore, in vivo, experiments with HCT116 xenografts in nude mice showed that the combination therapy inhibited tumor growth more effectively than ADT‐OH and celecoxib alone. In summary, this work elucidates the potential of ADT‐OH combined with celecoxib as an effective combination therapy strategy for colorectal cancer patients.

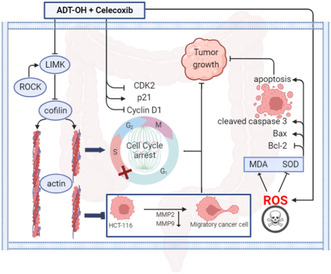

## 1 INTRODUCTION

Colorectal cancer (CRC) is currently ranked as the third most widespread cancer in the world and the second most common cause of cancer death, with the highest incidence among people aged 40–50, and the patients tend to be younger.^1^, ^2^ The occurrence rate of young people is increasing year by year, which is closely related to changes in lifestyle and dietary habits, as well as chronic colorectal inflammation, intestinal microbes, genetics, and mentality.^3^ Colorectal cancer occurs most frequently in the rectum, followed by the sigmoid colon, cecum, ascending colon, and descending colon.^4^, ^5^ In addition to the common operation, radiotherapy, and chemotherapy, the existing treatment methods for colorectal cancer also include immunotherapy, traditional Chinese medicine, and comprehensive treatment.^6^, ^7^ However, the early symptoms of colorectal cancer are insidious, generally asymptomatic, or not obvious, which is easy to be misdiagnosed and missed, and the local recurrence after treatment and poor prognosis leads to poor life quality of patients.^8^ Therefore, it is pressed for more beneficial methods to maximize the cure rate for colon cancer survivors.

Considerable reports support that cyclooxygenase 2 (COX‐2) levels are elevated during most colorectal cancer inflammation, suggesting that COX‐2 perhaps be involved in colorectal cancer inflammation and progression.^9^ In addition, certain reports suggest that COX‐2 is abnormally highly expressed in multitudinous human premalignant, malignant, and metastatic epithelial tumors including colorectal cancer.^10^ Celecoxib is a new generation of nonsteroidal anti‐inflammatory drugs (NSAIDs), which can selectively suppress the expression of COX‐2, thereby inhibiting the production of prostaglandins and achieving anti‐inflammatory and analgesic effects. At present, celecoxib has been authorized by the FDA for the clinical therapy of many kinds of arthritis and acute or chronic pain.^11^, ^12^ For the past few years, more and more studies have been conducted on the antitumor effects of celecoxib. Most researches remain in the preclinical stage and focus on elucidating the mechanism of effect. However, celecoxib is already in clinical trials for colorectal cancer,^13^ lung cancer,^14^ gastric cancer,^15^ and breast cancer.^16^ Although preclinical and clinical researches on celecoxib have shown viable effects in the prevention and therapy of cancer, the clinical application is still limited due to concerns about safety and potential for serious toxicity.

Among them, the most common is NSAIDs can induce gastroenteropathy.^17^ Studies have shown that NSAIDs inhibit endogenous H_2_S synthesis by decreasing the expression of cystathionine γ‐lyase (CSE). The additional decrease in H_2_S synthesis presumably led to an expansion of leukocyte adherence sequentially, which can cause gastric damage after the use of NSAID.^18^ Moreover, exogenous H2S‐repressed NSAID induced the expression of tumor necrosis factor α(TNFα), granulocyte infiltration, and the expression of endothelial and leukocyte adhesion molecules.^17^ It has subsequently been reported that the co‐administration of an H_2_S donor with NSAIDs inhibits NSAID‐induced leukocyte adherence and alleviates gastric injury.^19^, ^20^

ADT‐OH, an extensively used H_2_S donor, is a compound with the group structure of 3H‐1,2‐dithiole‐3‐thione.^21^ Compared with traditional H_2_S donors, ADT‐OH can stabilize H_2_S levels through slow release. Current research showed that, as the third important gas‐regulating molecule after CO and NO,^22^, ^23^ H_2_S is also involved in the regulation of tumor neogenesis and development, such as affecting cell cycle, angiogenesis, and apoptosis,^24^, ^25^ but only limited to preclinical studies. For example, ADT‐OH could inhibit the EMT progression in melanoma cells by inhibiting the CSE/CBS and FAK signaling pathways,^26^ and induce growth inhibition in human breast cancer cells by suppressing the PI3K/AKT/mTOR and RAS/RAF/MEK/ERK signal transduction.^27^

In cancer treatment, the drug combination strategy is becoming a common approach because it can demonstrate more effective antitumor effects while reducing the dose of each drug individually.^28^, ^29^ Therefore, we considered combining ADT‐OH with celecoxib, hoping to achieve better treatment of colorectal cancer while the addition of exogenous H_2_S would reduce the effect of celecoxib to induce gastrointestinal injury. In this study, our results demonstrated that ADT‐OH combined with celecoxib synergistically suppresses the proliferation and migration of human colorectal cancer HCT116 cells, alters the cell cycle and cytoskeleton, increases intracellular ROS, and promotes cell apoptosis. Furthermore, in vivo data demonstrated that the combination therapy strategy suppresses tumor growth more effectively than ADT‐OH and celecoxib alone, in HCT116 xenograft‐bearing nude mice. In conclusion, our work affords a potential combination therapy method for patients with colorectal cancer.

## 2 MATERIALS AND METHODS

### 2.1 Cells, cell culture, and reagents

Human normal colon epithelial cell line NCM460 was obtained from the American Type Culture Collection (ATCC). Human colorectal cancer cell line HCT116 and mouse colorectal cancer cell line CT26 were frozen in our laboratory. NCM460 and HCT116 were cultured in Dulbecco's Modified Eagle Medium (DMEM; Invitrogen) added 10% (v/v) fetal bovine serum (FBS; Invitrogen), 100 μg/mL streptomycin and 100 U/mL penicillin (Invitrogen, Carlsbad, CA, USA). And CT26 was grown in RPMI1640 medium supplemented with 10% (v/v) FBS, 100 μg/mL streptomycin, and 100 U/mL penicillin. All cells were cultured at 37°C in a humidified incubator with 5% CO_2_.

ADT‐OH and celecoxib were purchased from Shanghai Shifeng Biological Technology Co., Ltd. Cell Counting Kit‐8 (C0039) and One Step TUNEL Apoptosis Assay Kit (C1086) were purchased from Vazyme. And the BCA Protein Assay Kit (CW0014S) was purchased from CoWin Biosciences.

### 2.2 Cell proliferation assay

CCK8 was applied to assess the influence of ADT‐OH, celecoxib, or the combination method on cell proliferation. When NCM460, HCT116, and CT26 cells were cultured to the exponential growth phase, the cells were collected and adjusted to a concentration of 2 × 10^4^ cells/mL. Cells were then transferred to six replicate wells of 96‐well plates at a concentration of 100 μL/well and cultured overnight at 37°C. ADT‐OH (0, 7.5, 15, 30, 60, and 120 μM) and/or celecoxib (0, 7.5, 15, 30, 60, and 120 μM) were supplemented to the medium, and cells were then continued to grow 48 h. We calculated the IC_50_ of the two drugs against normal colon epithelial cell line NCM460 and colon cancer cell lines HCT116 and CT26 by using this gradient concentration treatment to obtain appropriate concentrations. The medium was removed after the treatment, and the mixture of 100 μL CCK8 and DMEM (1:100) was added into each well, and the culture was continued at 37°C for 1–2 h. Then the absorbance of 450 nm wavelength was detected by an enzyme labeling instrument, and the reference wavelength was 550 nm.

### 2.3 Cell cycle analysis

HCT116 and CT26 cells were treated with 30 μM of ADT‐OH, 30 μM of celecoxib, or the combination method for 48 h. The drug‐treated cells were digested using trypsin and cell precipitates were collected by centrifugation in a refrigerated centrifuge for 4 min (4°C, 800 rpm), and cell precipitates were cleaned twice with PBS. Cells were then slowly dripped with 70% pre‐cooled ethanol into the centrifuge tube and held overnight at 4°C. The fixed cells were washed and then stained with PI (2 mg/mL) in PBS containing RNase A (0.1 mg/mL) for 30 min at room temperature in the dark. FACSCalibur flow cytometry and CellQuest software (BD Biosciences) was used to analyze cell distribution with different DNA content at 530 nm excitation wavelength.

### 2.4 Cytoskeleton staining

HCT116 cells were seeded on sterilized slips for culture. When the cell density reached 40%–50% compatibility, 30 μM of ADT‐OH, 30 μM of celecoxib, or the combination method were added and continued to culture for 48 h. After 3 washes with ice PBS, the cell precipitate was resuspended in pre‐cooled 4% paraformaldehyde and fixed at 4°C overnight. The fixative was then centrifuged and rinsed with pre‐cooled PBS to remove excess fixative, then drilled with 0.5% Triton X‐100 and incubated at room temperature for 2 h. The residual solvent was washed with ice PBS, then the anti‐F‐Actin/FITC (fluorescein isothiocyanate) diluent (diluted with PBS containing 1% BSA, 1:40) was stained in the dark for 40 min, and 2‐(4‐Amidinophenyl)‐6‐indolecarbamidine dihydrochloride (DAPI) staining solution (Beyotime Biotechnology) was used for 2 min. Finally, 20 μL of the anti‐fade mounting medium was slowly added to the coverslip drip by drop, and the cytoskeletal morphology was observed and photographed under the fluorescence microscope.

### 2.5 Wound‐healing assay

HCT116 cells were inoculated in 6‐well culture plates. When the cell density reached 90%, the serum was starved for 12 h, and then the monolayer cells were vertically scraped with a sterile P‐200 micropipette to form the wound. The wells were gently rinsed three times with PBS to remove suspended cells and then continued to be cultured in DMEM containing 10% FBS with ADT‐OH (30 μM), celecoxib (30 μM), or the combination method for 48 h. The wound healing was observed with microphotographs of 10× magnification taken with an optical microscope (Carl Zeiss Axioplan 2) at 0, 24, and 48 h, respectively.

### 2.6 Transwell assay

HCT116 cells grown to the logarithmic growth phase were starved for 12 h. Cells were digested with trypsin, washed once with PBS, and then resuspended in serum‐free DMEM containing 30 μM of ADT‐OH, 30 of μM celecoxib, or both, respectively, to adjust the cell concentration to 6 × 10^5^ cells/mL. Then 200 μL cell suspension with a concentration of 5 × 10^5^ cells/mL was added into the upper cavity of the chamber, and 600 μL DMEM containing 10% FBS was supplemented into the lower cavity. After incubating in the cell incubator for 48 h, the culture medium in the upper chamber was carefully removed and the cells on the upper surface of the compartment membrane were softly wiped with a cotton swab. The chamber was fixed at room temperature in 4% paraformaldehyde for 30 min and then stained with 1% crystal violet and 2% ethanol in 100 mM borate buffer (pH 9.0) for 30 min. The number of cells that migrated to the submembrane surface was photographed at five fields under a microscope at a magnification of 100 ×.

### 2.7 Measurement of ROS

When HCT116 or CT26 cells in the six‐well plate grew to approximately 50% density, they were given 30 μM of ADT‐OH and/or 30 μM of celecoxib, respectively, and continued to culture for an additional 48 h. In a dark environment, DCFH‐DA was diluted to 10 μM with serum‐free DMEM at a ratio of 1:1000 on a clean bench. After slightly washing the cells twice with PBS, an appropriate amount of diluted DCFH‐DA solution was added to the well plate, incubating for 20 min in the dark in a constant temperature cell incubator at 37°C. Then, cells were washed 3 times using serum‐free DMEM to remove the DCFH‐DA that did not enter the cells, and then the intracellular ROS level was observed under a microscope. In addition, we also trypsinized and collected the cells, washed them with pre‐cooled PBS 3 times, and then detected the intracellular ROS levels using flow cytometry.

### 2.8 Measurement of malondialdehyde (MDA)

After being treated with 30 μM of ADT‐OH and/or 30 μM of celecoxib for 48 h, cells were digested using trypsin. The cells were collected into 1.5 mL centrifuge tubes and cell precipitation was washed with PBS. Subsequently, the cell pellet was resuspended in 1 mL of lysis buffer and sonicated to lyse the cells. After sufficient lysis, the supernatant was centrifugation at 4000 rpm for 15 min to obtain cell precipitation. The intracellular MDA level was detected by the MDA detection kit (Nanjing Jiancheng Bioengineering Institute), and the absorbance value of the sample at 532 nm was detected by a microplate reader.

### 2.9 Measurement of superoxide dismutase (SOD)

HCT116 cells were treated with 30 μM of ADT‐OH and/or 30 μM of celecoxib for 48 h and cell precipitate was collected. The cells were then resuspended in 0.5 mL PBS and the supernatant was collected after sonication. The SOD detection kit was used for detection in strict accordance with the instructions, and the absorbance was detected at 550 nm with a microplate reader. Calculation method: total SOD activity $\begin{array}{c} \left(U/\mathrm{mL}\right)=\left(\text{control}\;\mathrm{OD}\;\text{value}-\text{measured}\;\mathrm{OD}\;\text{value}\right)/\text{control}\;\mathrm{OD} \end{array}$ $\begin{array}{c} \text{value}÷50\%\times \text{dilution ratio of the reaction system}\times \;\text{dilution} \end{array}$ $\begin{array}{c} \text{ratio of the sample before testing} \end{array}$.

### 2.10 Detection of cell apoptotic

Cells were grown in 6‐well plates for 48 h in DMEM containing 10% FBS in the presence or absence of 30 μM of ADT‐OH and 30 μM of celecoxib alone or in combination. After the cells were digested, the original medium was discarded by centrifugation, and cells were washed with ice PBS three times. Then, cell precipitates were resuspended with HEPES buffer and stained with annexin V‐FITC (1 mg/mL) in the dark for 20 min on ice. Propidium iodide (20 μg/mL) was added to each sample tube before flow cytometry (BD Biosciences). Quantitative analysis of apoptotic cells was processed using CellQuest software (BD Biosciences).

### 2.11 RNA extraction and quantitative real‐time PCR

The total RNA of cell and tissue samples was extracted using Trizol reagent (Vazyme, R401‐01) in strict accordance with the instructions. Then, cDNA was performed using a ReverTra Ace® qPCR RT kit (Toyobo). Real‐time quantitative polymerase chain reaction (qPCR) was processed on a StepOne Real‐Time PCR system (Applied Biosystems) with AceQ® qPCR SYBR® Green Master Mix (Vazyme). Detailed sequences of primer used for qPCR are shown in Table S1. The expression level of detected genes in each sample was normalized to the expression level of actin mRNA. The data were analyzed using StepOne 2.1 software (Applied Biosystems) in strict accordance with the instructions.

### 2.12 Western blotting assay

Cell precipitates were collected after treatment and protein samples were obtained by whole‐cell lysis (Beyotime). Protein concentration was determined by the BCA reagent. Protein samples from different treatment groups were separated by electrophoresis in 12% SDS‐PAGE gels and electro‐transferred to PVDF membranes. After blocking with PBST containing 5% skim milk in a shaker at room temperature for 1 h, membranes were then incubated with primary antibodies: CDK‐2, p21, Cyclin D1, Cofilin, Bcl‐2, Bax, Cleaved Caspase‐3, β‐actin, and Tubulin (The detailed antibody information is listed in the following table.) at 4°C overnight. Following 3 washes in PBST, membranes were blotted with suitable secondary antibodies in a shaker at room temperature for 1 h. After 3 washes in PBST, the reactive blots were visualized by enhanced ECL detection reagents (Tanon). All tests were done in triplicate and processed at least 3 times independently.

**Table:** 

Antibody	Catalog number	Vendor
CDK‐2	18,048	Cell Signaling Technology
p21	2947	Cell Signaling Technology
Cyclin D1	55,506	Cell Signaling Technology
Cofilin	5175	Cell Signaling Technology
Bcl‐2	15,071	Cell Signaling Technology
Bax	ab32503	Abcam Inc.
Cleaved Caspase‐3	9661	Cell Signaling Technology
β‐Actin	3700	Cell Signaling Technology
Tubulin	5666	Cell Signaling Technology

### 2.13 Animals

Normal female athymic nude mice (5–6 weeks) were purchased from Changzhou Cavens Laboratory Animal Co., Ltd. and maintained under specific pathogen‐free (SPF) conditions for 1 week before the experiments. All the animal experiments involved in this work were approved by the Animal Care and Use Committee of Nanjing University with the approval number IACUC‐2109005. We conducted experiments in strict accordance with the recommendations of the Animal Protection Committee of Nanjing University guidelines.

### 2.14 In vivo xenograft tumor model of human HCT116 cell line

HCT116 cells (2 × 10^6^ cells in 100 μL) were injected subcutaneously into the right axilla of nude mice. The tumor volume was calculated by measuring the two maximum vertical diameters of the tumor using a vernier caliper on alternate days. When the tumor grew to a volume greater than 50 mm^3^, nude mice with similar tumor volumes were selected and randomly assigned into 4 groups: the control group, the ADT‐OH group, the celecoxib group, and the combination group. The mice in the ADT‐OH group or the combination group were injected intraperitoneally (i.p.) with ADT‐OH (37.5 mg/kg body weight) dissolved in 100% DMSO on alternate days. And the mice in the celecoxib group or the combination group were injected intraperitoneally (i.p.) with celecoxib (50 mg/kg body weight) dissolved in 100% DMSO on alternate days. Mice in the control group received an i.p. injection of equal doses of DMSO. The treatment lasted for 21 days, and the nude mice were euthanized when tumors in the model group grew to a volume of approximately 1500 mm^3^. Subcutaneous tumor, liver, kidney, and spleen of nude mice were dissected. During the treatment, tumor volume was detected daily, and the body weight was monitored.

### 2.15 Hematoxylin and Eosin (H&E) staining and TUNEL assays

After 21 days of treatment, the tumor‐bearing mice were sacrificed, and the tumor tissue was carefully removed. The tumor tissue was fixed and embedded and prepared into 3 μM sections by H&E staining scheme. Terminal deoxynucleotidyl transferase dUTP Notch end labeling (TUNEL) tests were processed using the TUNEL BrightGreen apoptosis detection kit, according to the kit's operating instructions (Vazyme).

### 2.16 Statistical analysis

The experiments were conducted at least 3 times, and the results were broadly similar. The results were displayed as the mean ± standard deviation (SD) after the analyses were accomplished using Graph Pad Prism 8.0 (Graph Pad Software). To analyze the difference between the two groups, we used a two‐tailed Student's *t*‐test. And when analyzing the significance between groups, we used the One‐way analysis of variance (ANOVA). *p < 0.05* was regarded as statistically significant in our analyses.

## 3 RESULTS

### 3.1 ADT‐OH combined with celecoxib inhibits HCT116 cell proliferation

**FIGURE 1:**
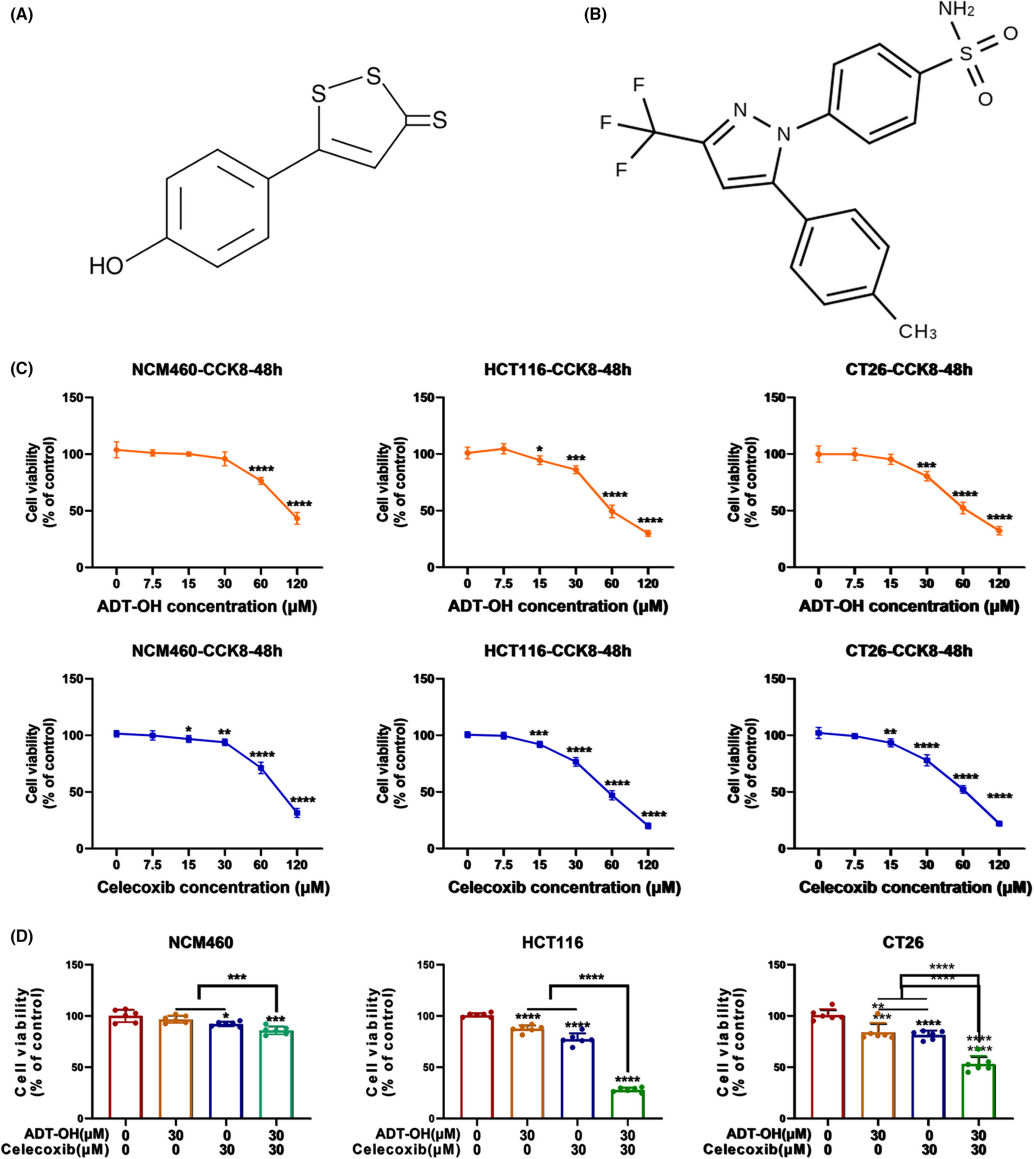
ADT‐OH combined with celecoxib inhibits HCT116 cell proliferation. (A) Chemical structure of ADT‐OH. (B) Chemical structure of celecoxib. (C) NCM460, HCT116, and CT26 cells were treated with different concentrations of ADT‐OH or celecoxib for 48 h, and the cell proliferation ability was calculated by the CCK‐8 method. (D) After NCM460, HCT116, and CT26 cells were treated with ADT‐OH (30 μM) and/or celecoxib (30 μM) for 48 h, the cell proliferation ability was detected by the CCK‐8 method. All tests were set up with 6 multiple holes and performed 3 times independently. Data are presented as mean ± SD. *p < 0.05, **p < 0.01, ***p < 0.001, ****p < 0.0001.

The chemical structure of ADT‐OH is displayed in Figure 1A. Our previous studies have shown that ADT‐OH releases H_2_S slowly in cells in a time‐ and concentration‐dependent manner.^26^, ^30^ The chemical structure of celecoxib is displayed in Figure 1B. Cell proliferation was detected by CCK8 assay. We found that both ADT‐OH and celecoxib inhibited the proliferation of colorectal cancer cell lines HCT116 (human) and CT26 (mouse) in a concentration‐dependent manner (Figure 1C). After the treatment with ADT‐OH for 48 h, ADT‐OH showed an IC_50_ value of 63.63 μM against HCT116 cells and an IC_50_ value of 67.81 μM against CT26 cells, while the IC_50_ value against NCM460 was 115.59 μM. Similarly, After the treatment with celecoxib for 48 h, celecoxib showed an IC_50_ value of 52.05 μM against HCT116 cells and an IC_50_ value of 56.21 μM against CT26 cells, while the IC_50_ value against NCM460 was 79.63 μM (Table S2). Noteworthy, high doses of ADT‐OH and celecoxib also inhibited the proliferation of human normal colonic epithelial cell line NCM460 (Figure 1C). Therefore, we selected the lowest dose that significantly inhibited the proliferation of HCT116 and CT26 cells but showed only a slight effect on NCM460 for the next combination therapy. Our data suggested that the lowest concentration of the combination (30 μM ADT‐OH + 30 μM celecoxib) for 48 h produced significant proliferation inhibition on HCT116 and CT26 cells, but only had a slight proliferation inhibition on NCM460 (<20%) (Figure 1D). Thus, we chose these concentrations of the combination therapy in the subsequent experiments.

### 3.2 ADT‐OH combined with celecoxib causes cell cycle arrest at the G0/G1 phase in HCT116 cells

**FIGURE 2:**
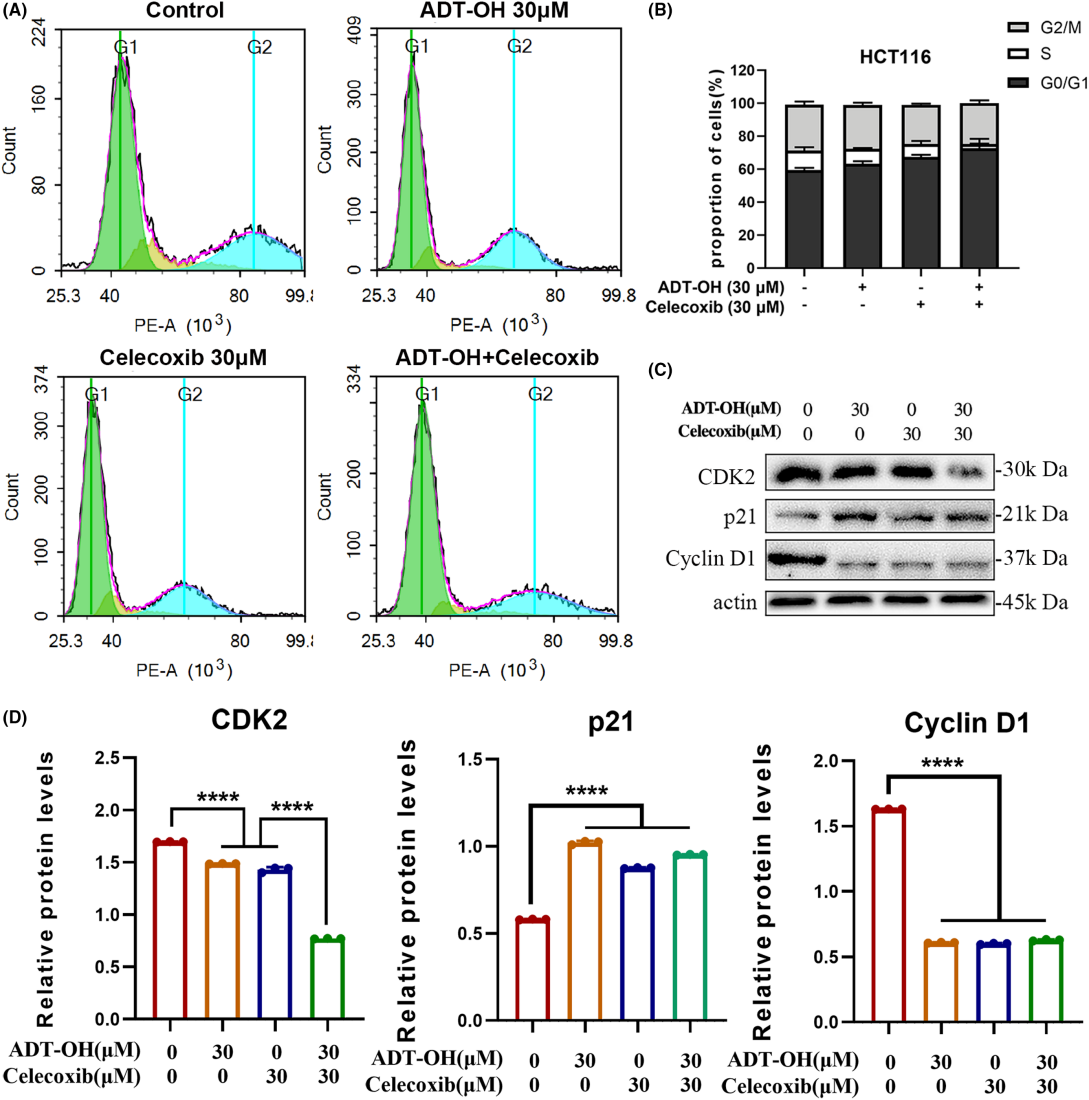
ADT‐OH combined with celecoxib causes cell cycle arrest at the G0/G1 phase in HCT116 cells. (A) The cell cycle distribution of HCT116 cells was detected by flow cytometry after 48 h of treatment with ADT‐OH (30 μM) and/or celecoxib (30 μM). (B) Statistics of cell cycle distribution. (C) Detection of cell cycle‐related protein expression levels by western blotting. (D) Statistics of cell cycle‐related protein expression levels. All tests were done in triplicate and performed 3 times independently. Data are presented as mean ± SD. ****p < 0.0001.

Previous researches have suggested that celecoxib can exert its anti‐tumor effect by inhibiting cell proliferation.^31^ Consequently, we detected the effectiveness of ADT‐OH, celecoxib, or their combination on cell cycle distribution. HCT116 and CT26 cells were treated with 30 μM of ADT‐OH, 30 μM of celecoxib or their combination, respectively. After 48 h, the treated cells were harvested and fixed, and the distribution of the cell cycle was detected. As displayed in Figure 2A,B, compared with the control group, both 30 μM of ADT‐OH and 30 μM of celecoxib increased the percentage of HCT116 cells in the G0/G1 phase to a certain extent. After the combination of the two drugs, the percentage of cells in the G0/G1 phase upregulated more significantly. Thus, more cells were arrested in the G0/G1 phase. It was shown that ADT‐OH combined with celecoxib could change the cell cycle of HCT116 cells through arresting cells in the G0/G1 phase. Consistent with this, we found that ADT‐OH combined with celecoxib also resulted in more CT26 cells being arrested in the G0/G1 phase (Figure S1A,B).

To explore the mechanism by which ADT‐OH combined with celecoxib changes the cell cycle of HCT116, we treated cells with ADT‐OH, celecoxib, or their combination for 48 h. Then we extracted total intracellular protein and analyzed the expression levels of G1 phase‐related proteins by western blots, such as cyclins CDK2, p21, and cyclin D1. Compared with the control group, the expression level of CDK2 protein was significantly reduced when treated with ADT‐OH or celecoxib alone. In addition, when cells were treated with ADT‐OH combined with celecoxib, the CDK2 protein displayed a more remarkable decline in the expression level. The expression level of p21 protein was significantly upregulated after administration, while that of Cyclin D1 was significantly downregulated, but the expression of the two molecules did not show the synergistic effect of ADT‐OH and celecoxib (Figure 2C,D). Therefore, we concluded that ADT‐OH combined with celecoxib altered the cell cycle of HCT116 cells by synergistically reducing the expression level of CDK2 protein, thereafter more cells were arrested in the G0/G1 phase.

### 3.3 ADT‐OH combined with celecoxib alters the cytoskeleton of HCT116 cells

**FIGURE 3:**
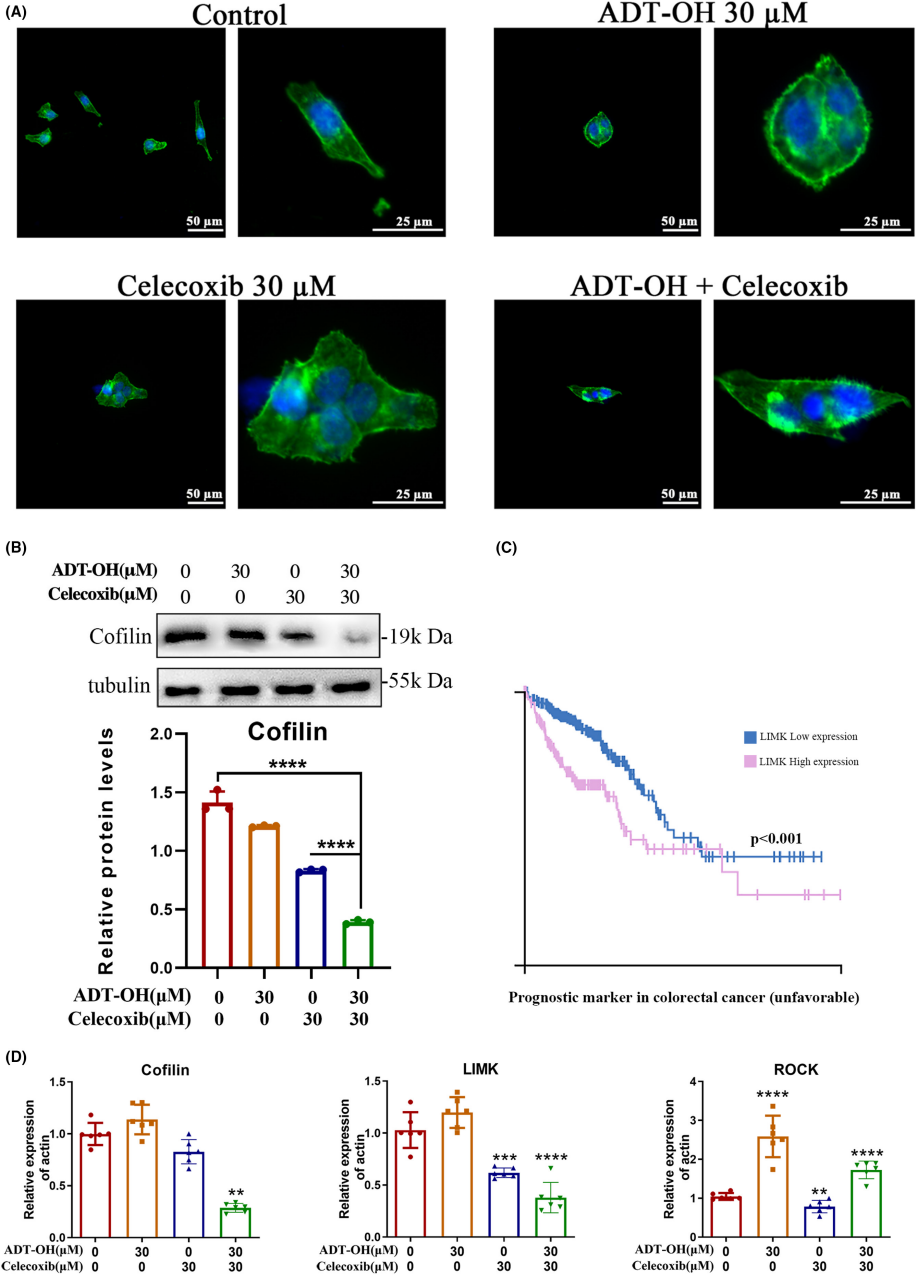
ADT‐OH combined with celecoxib alters the cytoskeleton of HCT116 cells. (A) The immunofluorescence was carried out to display F‐actin (anti‐F‐actin/FITC, green), and nuclei (DAPI, blue) in HCT116 cells after 48 h of treatment with ADT‐OH (30 μM) and/or celecoxib (30 μM). (B) Western blot was used to detect the protein expression level of cofilin. (C) High expression of LIMK is a negative prognostic indicator of colorectal cancer. (D) RNA expression levels of cofilin, LIMK, and ROCK. All tests were done in triplicate and performed 3 times independently. Data are presented as mean ± SD. **p < 0.01, ***p < 0.001, ****p < 0.0001.

Furthermore, in order to elucidate the impact of the drugs on the actin cytoskeletons of HCT116 cells, a phalloidin immunofluorescence staining was conducted after cells were treated with ADT‐OH, celecoxib, or both. When compared to the control, the edges of cells became blurred after 48 h of treatment with ADT‐OH or celecoxib alone, and filamentous and fascia actin skeleton fibers appeared around the cells. This was even more pronounced in cells treated with ADT‐OH combined with celecoxib: the intact edges of cells were lost and replaced by distinct filamentous, fascicled actin skeletal fibers (Figure 3A). Therefore, ADT‐OH combined with celecoxib could significantly alter the cytoskeleton morphology.

Studies have shown that cofilin is a dynamic regulator of actin/tubulin.^32^ Thus, we measured the expression level of cytoskeleton‐related protein cofilin by western blot. The data suggested that the protein level of cofilin was significantly decreased under ADT‐OH or celecoxib monotherapy, and was further downregulated by the combination of the two drugs (Figure 3B). In addition, the Human Protein Atlas database showed that cofilin and its upstream kinase LIMK protein are overexpressed in multiple tumors containing colorectal cancer. Besides, LIMK is an unfavorable prognostic indicator for colorectal cancer (Figure 3C; Figure S2A–D). We then examined the mRNA expression levels of cofilin and its upstream kinase LIMK, as well as the upstream kinase ROCK.^33^ The results suggested that ADT‐OH combined with celecoxib could significantly reduce the mRNA levels of cofilin and LIMK (Figure 3D). It is demonstrated that ADT‐OH combined with celecoxib could alter the cytoskeleton of HCT116 cells by reducing the expression of cofilin and LIMK.

### 3.4 ADT‐OH combined with celecoxib inhibits HCT116 cell migration

**FIGURE 4:**
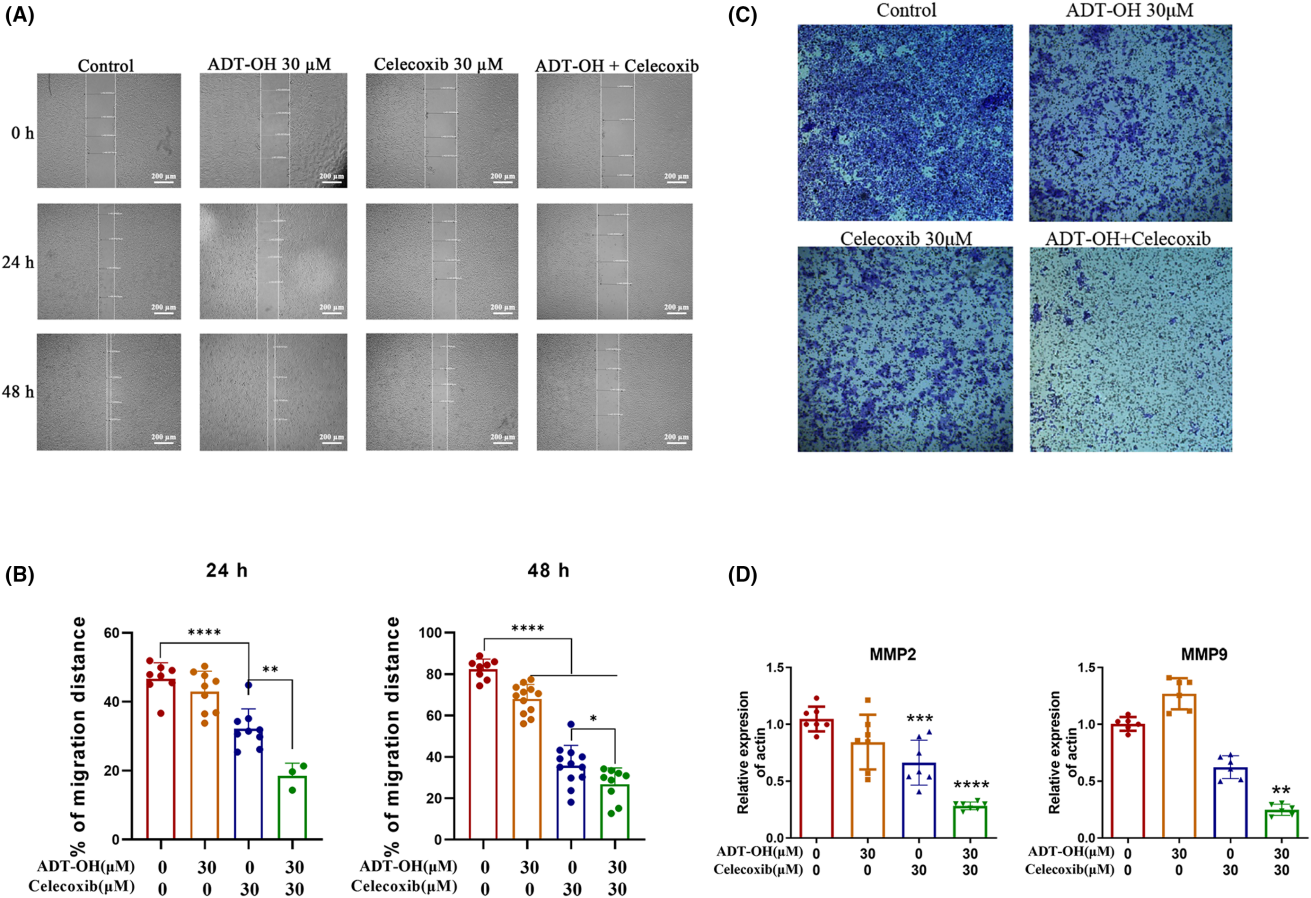
ADT‐OH combined with celecoxib inhibits HCT116 cell migration. (A) HCT116 cells were treated with ADT‐OH (30 μM) and/or celecoxib (30 μM) for 48 h. Photos were taken at 0, 24, and 48 h after scratch creation, respectively. The images shown are representative of three independent experiments. (B) Statistics of cell migration ability. (C) HCT116 cells were treated with ADT‐OH (30 μM) and/or celecoxib (30 μM) for 48 h, and cells migrating to the lower membrane were stained and counted in five fields. (D) mRNA expression levels of MMP2 and MMP9. All tests were done in triplicate and performed 3 times independently. Data are presented as mean ± SD. *p < 0.05, **p < 0.01, ***p < 0.001, ****p < 0.0001.

To further observe the influence of ADT‐OH and celecoxib on tumor cell migration, wound healing, and transwell tests were processed. After 24 h of drug treatment, compared with the control group, 30 μM of ADT‐OH or 30 μM of celecoxib alone could inhibit scratch healing, while ADT‐OH combined with celecoxib could significantly slow down the healing speed, and the healing area was only 20% of the control group. At 48 h, the healing area ratios of the scratches in the ADT‐OH and celecoxib treatment groups were about 70% and 40% of those in the control group, respectively, while the healing ratio in the combination treatment group was only about 25% of that in the control group (Figure 4A,B). The longitudinal migration ability of HCT116 cells was detected by transwell assay. As shown in Figure 4C, after treatment with ADT‐OH or celecoxib alone, the cell infiltration of HCT116 cells was significantly inhibited, while the combination of the two drugs showed a lower number of cells that successfully penetrated the membrane. In general, these results suggested that ADT‐OH combined with celecoxib could inhibit the migration of HCT116 cells. The matrix metalloproteinase family (MMP) is a Zn^2+^/Ca^2+^‐dependent protease family that plays a critical role in tumor metastasis and invasion. The expression of MMP2 and MMP9 is related to the progression of colorectal cancer.^34^ Thus, the mRNA expression levels of migration‐related proteins MMP2 and MMP9 were measured. Compared with the control group, the mRNA levels of MMP2 and MMP9 were significantly reduced after the treatment of ADT‐OH combined with celecoxib (Figure 4D). Overall, these data demonstrated that ADT‐OH combined with celecoxib could suppress the migration of HCT116 cells by inhibiting the expression of MMP2 and MMP9.

### 3.5 ADT‐OH combined with celecoxib promotes ROS production in HCT116 cells

**FIGURE 5:**
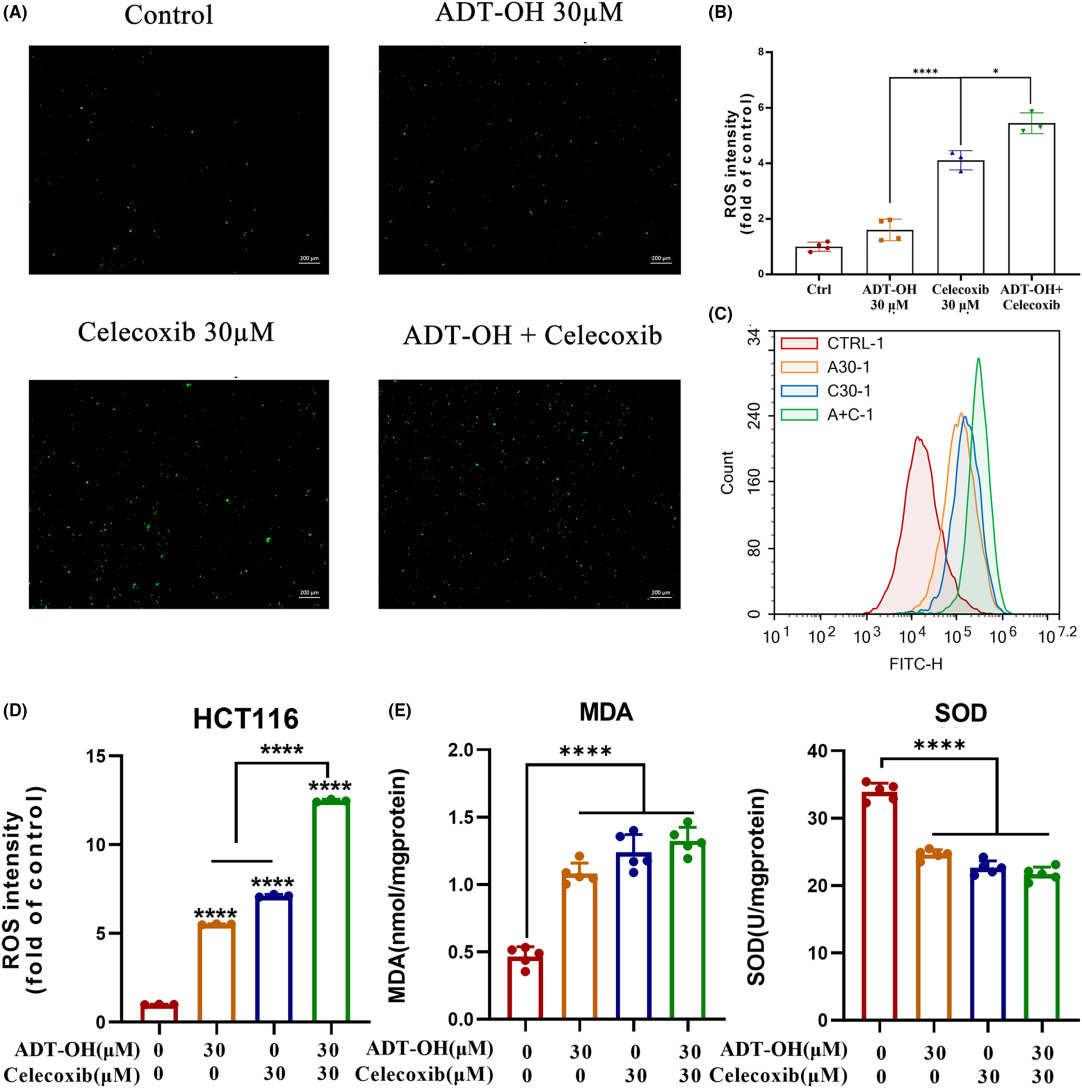
ADT‐OH combined with celecoxib promotes ROS production in HCT116 cells. (A) ROS levels in HCT116 cells were observed under the microscope after 48 h of treatment with ADT‐OH (30 μM) and/or celecoxib (30 μM). (B) Statistics of ROS levels in HCT116 cells with different drug treatments. (C) HCT116 cells were treated with ADT‐OH (30 μM) and/or celecoxib (30 μM) for 48 h, and flow cytometry was used to detect the level of ROS. (D) Statistics of ROS levels in HCT116 cells with different drug treatments. (E) MDA and SOD levels in HCT116 cells were detected according to the manufacturer's instructions after 48 h of treatment with ADT‐OH (30 μM) and/or celecoxib (30 μM). All tests were done in triplicate and performed 3 times independently. Data are presented as mean ± SD. *p < 0.05, ****p < 0.0001.

Previous studies have shown that celecoxib can induce cancer cell death by increasing ROS generation.^35^ After 48 h of ADT‐OH or celecoxib treatment alone or in combination, the ROS levels in HCT116 cells were detected by microscope or flow cytometry. Compared with the control group, 30 μM of ADT‐OH or celecoxib alone could increase the level of intracellular ROS to some extent. And the ROS level increased the most significantly in the combined group (Figure 5A–D; Figure S3A). The results above showed that ADT‐OH combined with celecoxib could increase the level of ROS in HCT116 cells. Similarly, we observed a consistent pattern in CT26 cells (Figure S3B–D).

Elevated intracellular ROS levels are often accompanied by the accumulation of lipid peroxides such as MDA, because ROS can interact with polyunsaturated fatty acids on biofilms, causing lipid peroxidation.^36^, ^37^ In addition, SOD also plays a critical role in the oxidative and antioxidant balance of cells. Therefore, we used the kits to measure the content of MDA and SOD in HCT116 cells. Compared with the control group, HCT116 cells treated with ADT‐OH or celecoxib for 48 h showed a significant accumulation of intracellular lipid peroxide MDA, while the combination further promoted the level of MDA (Figure 5E). The intracellular SOD level was significantly decreased after 48 h treatment of cells with ADT‐OH or celecoxib alone, while ADT‐OH combined with celecoxib further reduced the SOD level (Figure 5E). Therefore, we speculated that ADT‐OH combined with celecoxib might aggravate cell damage by increasing the level of intracellular ROS, suppressing the ability of cells to scavenge oxygen free radicals, thus making cells further attacked by oxygen free radicals.

### 3.6 ADT‐OH combined with celecoxib promotes HCT116 cell apoptosis

**FIGURE 6:**
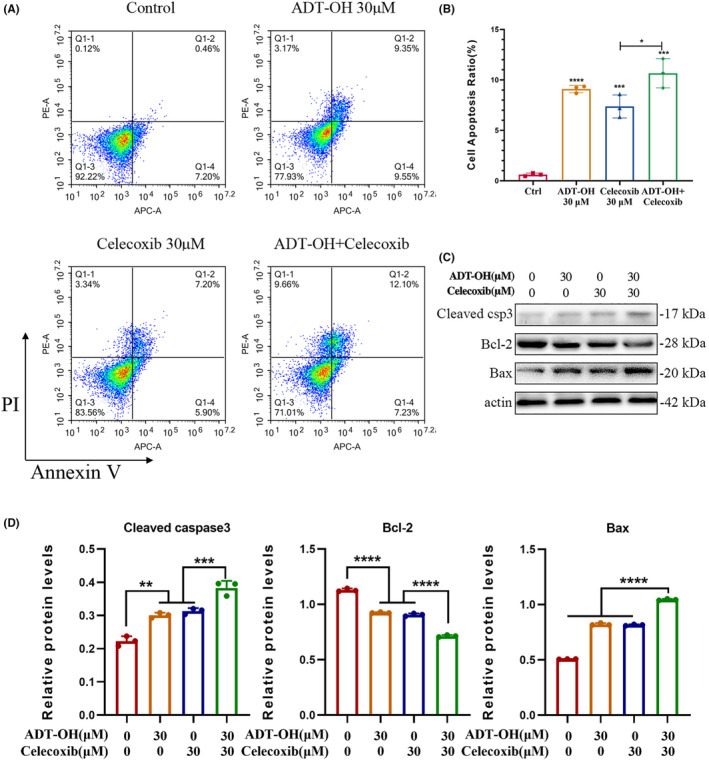
ADT‐OH combined with celecoxib promotes HCT116 cell apoptosis. (A) After 48 h of treatment with ADT‐OH (30 μM) and/or celecoxib (30 μM), the level of apoptosis of HCT116 cells was detected by flow cytometry. (B) Statistics of apoptosis levels in HCT116 cells with different drug treatments. (C) Detection of cell apoptosis‐related protein expression levels by western blotting. (D). Statistics of cell apoptosis‐related protein expression levels. All tests were done in triplicate and performed 3 times independently. Data are presented as mean ± SD. **p < 0.01, ***p < 0.001, ****p < 0.0001.

Elevated intracellular ROS levels tend to induce apoptosis.^38^ To verify the effect of ADT‐OH combined with celecoxib on HCT116 and CT26 cell apoptosis, flow cytometry detection was performed in our research. Our data suggested that compared with the control group, treatment with 30 μM of ADT‐OH or 30 μM of celecoxib alone could significantly increase the apoptotic ratio of both HCT116 and CT26 cells, and the combination method led to a more significant increase (Figure 6A,B; Figure S4A,B). Hence, ADT‐OH combined with celecoxib promoted the apoptosis of HCT116 cells.

Caspases cysteine protease is a key enzyme in apoptosis or programmed death. When the apoptosis program is initiated, caspase‐3 will be converted into activated cleaved caspase‐3.^39^ In addition, Bcl‐2 protein and Bax protein also play a critical role in the progress of apoptosis, among which Bcl‐2 is anti‐apoptotic, while Bax is pro‐apoptotic.^40^ To explore the mechanism of ADT‐OH combined with celecoxib to promote cell apoptosis, we measured the expression levels of apoptosis‐related proteins cleaved caspase‐3, Bcl‐2, and Bax through western blot. Our data suggested that, compared with the control group, ADT‐OH or celecoxib alone could significantly increase Bax and cleaved caspase‐3, and downregulate Bcl‐2. However, after administration of ADT‐OH combined with celecoxib, the expression of cleaved caspase‐3 and Bax was increased, and Bcl‐2 was decreased more significantly (Figure 6C,D). The results above showed that ADT‐OH combined with celecoxib could facilitate the apoptosis of HCT116 cells through upregulating the protein levels of cleaved caspase‐3 and Bax and downregulating that of Bcl‐2.

### 3.7 ADT‐OH combined with celecoxib retards the development of colon cancer xenografts in nude mice

**FIGURE 7:**
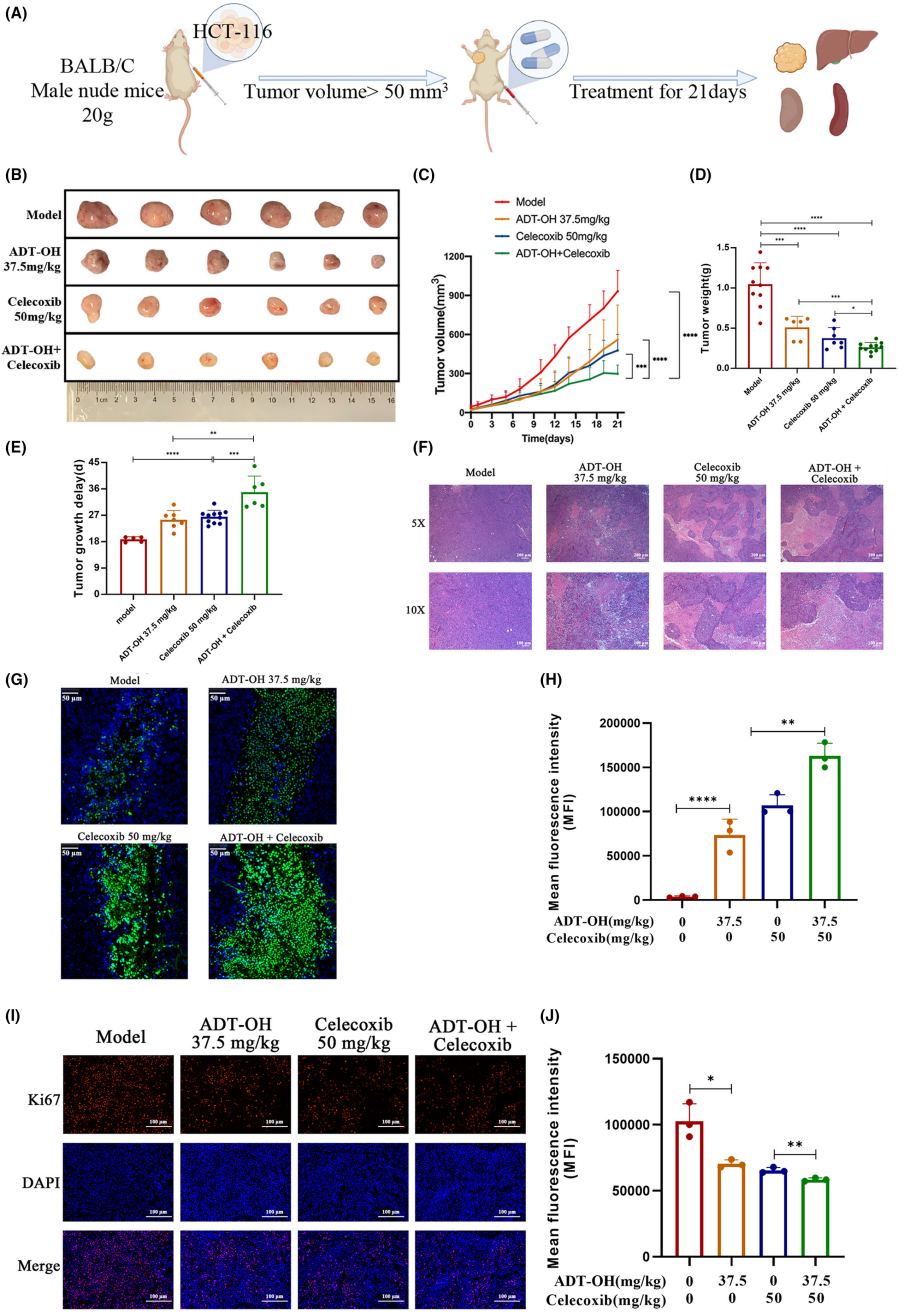
ADT‐OH combined with celecoxib retards the development of colon cancer xenografts in nude mice. (A) Schematic diagram of the animal experiment protocol. (B) Images of the excised tumors from each therapeutic group were shown. (C) Tumor volumes under different treatments were compared. (D) At the end of the study, the excised tumors from each group were weighed. (E) Tumor growth delay of each therapeutic group. (F) The necrosis changes of tumors receiving different treatments were observed by H&E staining. (G) TUNEL assay was used to detect apoptotic cells (stained green) (200×). (H) TUNEL‐positive cells were counted from three fields with the highest density of positive‐stained cells in each section to determine the percentage of apoptotic cells. (I) Representative pictures of Ki67 staining in tumor tissue. (J) Statistics of Ki67 fluorescence intensity in tumor tissue. Data are presented as mean ± SD. *n* = 6 mice in each group. *p < 0.05, **p < 0.01, ***p < 0.001, ****p < 0.0001.

To verify the in vivo effectiveness of the drug combination method with ADT‐OH and celecoxib on colon cancer, HCT116 cells were inoculated subcutaneously in nude mice. ADT‐OH and celecoxib were used alone or in combination for 21 days when the tumors grew to about 50 mm^3^ (Figure 7A). After 21 days of combined treatment, tumor volume was significantly decreased compared with mice treated with either ADT‐OH or celecoxib monotherapy (Figure 7B,C). Additionally, tumor weight was significantly lower in the combination group than in the ADT‐OH or celecoxib monotherapy group (Figure 7D). Tumor‐delayed growth time was further analyzed. Our data suggested that compared with the model group, the ADT‐OH or the celecoxib treatment group had significantly longer tumor growth delay time, but there was no remarkable discrepancy between the two groups. Compared with the monotherapy groups, the combination treatment significantly prolonged the delayed tumor growth time (Figure 7E). In conclusion, ADT‐OH combined with celecoxib could synergistically inhibit the growth of subcutaneous tumors in nude mice.

The paraffin sections of nude mice tumor tissue were further stained with H&E, and the necrosis of tumor tissue was observed under the microscope. Compared with the control group, ADT‐OH or celecoxib monotherapy significantly increased the area of tumor tissue necrosis, but the proportion increased more significantly after drug combination treatment (Figure 7F). Similarly, Ki67 staining suggested that the proportion of positive cells in the ADT‐OH combined with the celecoxib group was significantly lower than that in the model group and the single administration group (Figure 7G,H). TUNEL results suggested that ADT‐OH or celecoxib alone promoted the apoptosis rate in tumor tissues, while the apoptotic area was significantly upregulated in the combination treatment group (Figure 7I,J). The data above showed that ADT‐OH combined with celecoxib could synergistically suppress tumor cell proliferation and facilitate tumor cell death in vivo.

Additionally, there was no obvious variation in the body weight of nude mice during the whole therapy progress (Figure S5A). After the treatment, the livers, kidneys, and spleens of nude mice were dissected for morphological observation, weighing, and H&E staining. There was no obvious discrepancy in the shape and weight of the livers, kidneys, and spleens in each treatment group compared with the complete blank control group (Figure S5B,C). Except for the model group, there was no obvious discrepancy in the H&E staining of the liver and kidney pathological sections in other groups compared with the blank control group (Figure S5D). In general, all the data above demonstrated that ADT‐OH combined with celecoxib had no obvious toxicity and side effects in vivo at this combined dose.

## 4 DISCUSSION

CRC is currently the second most familiar cancer in females and the third most familiar in males, and it is one of the leading reasons for cancer‐related mortality, accounting for 9.2% of deaths worldwide.^41^, ^42^ At present, the therapies of CRC mainly include surgical resection,^43^ radiotherapy,^44^ chemotherapy,^45^ and immunotherapy.^7^ However, various treatment options have certain limitations, such as affecting nutrient absorption, local recurrence, and poor prognosis after surgical resection of adenomas. In addition, the non‐specificity of radiotherapy and chemotherapy also has a certain killing effect on normal cells, resulting in low life quality and shortened lifespan of patients after treatment.^46^ Therefore, there is an urgent need to seek more effective treatment options for colorectal cancer with less impact on patients' life after surgery.

COX‐2 is an important element in the occurrence and subsequent progression of colorectal cancer, and increased COX‐2 mRNA and protein levels have been detected in most colorectal cancers.^47^ Although the regulatory mechanisms of COX‐2 over‐expression in the pathogenesis of colorectal cancer have not been thoroughly investigated, the anticancer capability of both selective and non‐selective COX‐2 inhibitors has been demonstrated. Remarkably, celecoxib, a COX‐2 inhibitor with antitumor properties, has been reported for the treatment of colorectal cancer. For example, studies in the colon cancer cell line HT‐29 demonstrated the ability of celecoxib to exert anticancer/antitumor efficacy in vitro.^48^ Similar effects were obtained in our studies in the colorectal cancer cell line HCT116. Furthermore, the anticancer effect appears to be enhanced when celecoxib is combined with other anticancer agents.^49^ It is well acknowledged that H_2_S has been involved in multitudinous physiological response processes, such as anti‐inflammation,^50^ oxidative stress,^51^ vasoregulation,^52^ neuromodulation,^22^ post‐myocardial infarction reperfusion damage protection,^53^ and insulin resistance.^54^ Here, we used ADT‐OH as a common H_2_S sustained‐release donor, and studies have shown that H_2_S performs physiological effects at a broad concentration range (10–300 μM).^55^, ^56^ Studies also have reported that H_2_S is associated with various inflammatory progressions. In this context, H_2_S has physiological and pathophysiological roles, especially in the colon tissue, where endogenous bacteria generate H_2_S, creating an “H_2_S environment” in which parenchymal cells and associated immune cells are influenced, thereby regulating their cell viability and physiological function. Consistently, our physiology highlighted that ADT‐OH combined with celecoxib had a stronger inhibitory effect on the development of colorectal cancer in vitro and in vivo.

The formation and progression of cancers are the results of abnormal proliferation, apoptosis, and invasion of cancer cells, which are regulated by a variety of factors. Studies have shown that celecoxib has anti‐proliferation, anti‐migratory and pro‐apoptotic functions in various tumor cells.^57^ Likewise, an increasing number of studies have elucidated the numerous critical roles of H_2_S in tumor cells including inhibition of proliferation, prevention of metastasis and tumor angiogenesis, and induction of apoptosis.^58^, ^59^ Consistent with these reports, our study suggested that both ADT‐OH and celecoxib could effectively suppress the proliferation of HCT116 and CT26 cells, and the inhibitory effect was more significant after the combination. Cell proliferation is the foundation of organism growth, development, reproduction, and heredity, and is closely related to the normal progression of the cell cycle. Our data suggested that ADT‐OH combined with celecoxib induced HCT116 and CT26 cell cycle arrest in the G0/G1 phase mainly by downregulating CDK2 expression level.

Subsequently, we investigated the impact of the combination method on cytoskeleton and cell invasion. In light of our research, ADT‐OH combined with celecoxib significantly altered the cytoskeletal morphology. Cofilin is known to be a dynamic regulator of actin/tubulin,^32^ and the combination method significantly down‐regulates the expression level of cofilin compared with one drug alone. Likewise, the inhibitory effect on migration was also higher with the combination of the two drugs, as exhibited by significantly decreased expression levels of MMP2 and MMP9.

Previous reports have shown that celecoxib can induce cancer cell death by increasing the production of ROS.^35^ Consistent with this, our results suggested that both ADT‐OH and celecoxib could increase ROS levels in HCT116 cells to a certain extent, but the combination group showed the most significant increase in intracellular ROS levels. The mechanism of this process mainly involves promoting the accumulation of intracellular MDA and decreasing the level of intracellular SOD. It is wildly‐known that elevated intracellular ROS levels tend to induce apoptosis,^38^ and apoptosis is mainly accomplished by caspase‐3 via the regulation of various signaling pathways related to apoptosis. Our current data suggested that ADT‐OH combined with celecoxib facilitated apoptosis in HCT116 cells via activation of caspase‐3. In addition, three subfamilies of Bcl‐2 proteins are involved in apoptosis regulation: anti‐apoptotic Bcl‐2 proteins, pro‐apoptotic BH3‐only proteins, and pro‐apoptotic effector proteins.^60^ Here, the combined method also inhibited the expression of Bcl‐2 and facilitated the expression of Bax, indicating that ADT‐OH combined with celecoxib could activate the endogenous apoptosis signaling pathway.

**FIGURE 8:**
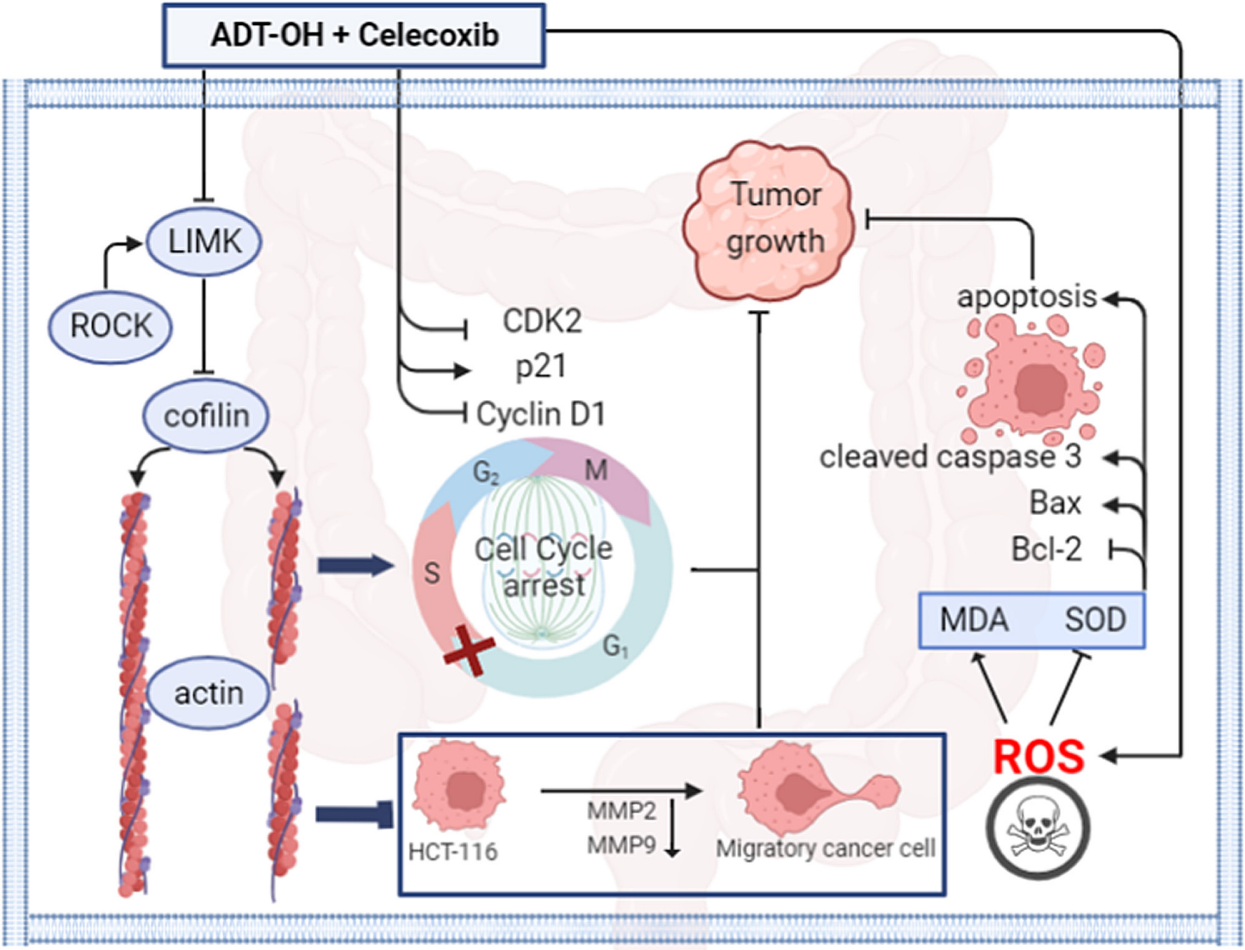
A working model of the synergistic effect of ADT‐OH and celecoxib on colorectal cancer HCT116 cells. In HCT116 cells, the combination of ADT‐OH and celecoxib inhibited cell migration by downregulating the expressions of LIMK, cofilin, MMP2, and MMP9. Furthermore, the combination of ADT‐OH and celecoxib resulted in cell cycle arrest in the G2/M phase by upregulating p21 expression and downregulating CDK2 and cyclin D1 expression. Meanwhile, the combination of ADT‐OH and celecoxib promoted the production of reactive oxygen species in HCT116 cells, which in turn led to the accumulation of intracellular MDA and the decrease of SOD. Furthermore, the combination of ADT‐OH and celecoxib induced apoptosis by upregulating cleaved caspase 3 and Bax expression and downregulating Bcl‐2 expression. All the above may explain the synergistic effect of ADT‐OH and celecoxib in inhibiting the growth of HCT116 cells.

Our current study is the first to report the therapeutic effect of ADT‐OH combined with celecoxib on colorectal cancer. We found that ADT‐OH combined with celecoxib synergistically inhibited the proliferation and migration ability of human colorectal cancer HCT116 cells, altered the cell cycle and cytoskeleton, increased intracellular ROS, and promoted cell apoptosis (Figure 8). We improve monotherapy by combining therapies that affect multiple signaling pathways. Among them, the extensive crosstalk between multiple pathways may account for a better therapeutic effect. Unfortunately, more efforts are urgently needed in the future to illuminate the specific conditions of these crosstalk, or to identify the key targets to help us better understand the specific mechanism of the combination therapy. Furthermore, considering the complexity of the animal, we also conducted in vivo experiments with HCT116 xenografts in nude mice. Our data suggested that the combination therapy inhibited tumor growth more effectively than ADT‐OH and celecoxib alone and no significant side effects were observed at the drug concentrations we used. In summary, this work elucidates the potential of ADT‐OH combined with celecoxib as an effective combination therapy strategy for colorectal cancer patients.

It is worth mentioning that there is limited information on H_2_S donors (such as in vivo metabolism and clearance), posing challenges to its applicability. To this end, it is critical to evaluate the concentration range of H_2_S donors and identify all possible toxic side effects and clearance processes in vivo. Besides, possible mutual crosstalk among different molecules, such as drug combinations or molecular hybrids, requires a supernumerary inquiry to obtain superior efficacy and lower toxic side effects. Further, modification of these two drugs, especially ADT‐OH, may be considered to further reduce their working concentration or achieve better therapeutic effects.

## AUTHOR CONTRIBUTIONS

**Huangru Xu:** Data curation (equal); formal analysis (equal); investigation (equal); methodology (equal); validation (equal); visualization (equal); writing – original draft (equal). **Ping Li:** Data curation (equal); formal analysis (equal); investigation (equal); methodology (equal); validation (equal); visualization (equal). **Hailin Ma:** Investigation (supporting); methodology (supporting); validation (supporting). **Yuanhao Tan:** Investigation (supporting); methodology (supporting); validation (supporting). **Xiaoyang Wang:** Investigation (supporting); methodology (supporting). **Fangfang Cai:** Investigation (supporting); methodology (supporting). **Jiaqi Xu:** Methodology (supporting). **Huisong Sun:** Methodology (supporting). **Hongqin Zhuang:** Conceptualization (lead); data curation (equal); funding acquisition (equal); project administration (equal); resources (supporting); supervision (lead); writing – review and editing (lead). **Zi‐Chun Hua:** Conceptualization (lead); funding acquisition (lead); project administration (lead); resources (supporting); supervision (lead); writing – review and editing (supporting).

## FUNDING INFORMATION

This study was supported in part by grants from the National Natural Sciences Foundation of China (82130106, 32250016), Nanjing Special Fund for Life and Health Science and Technology (202110016, China), Changzhou Bureau of Science and Technology (CJ20220019, China) and Jiangsu TargetPharma Laboratories Inc., China.

## CONFLICT OF INTEREST STATEMENT

The authors declare that the research was conducted in the absence of any commercial or financial relationships that could be construed as a potential conflict of interest.

## ETHICS APPROVAL AND CONSENT TO PARTICIPATE

Animal welfare and experimental procedures were performed in strict accordance with high standard animal welfare and other related ethical regulations approved by the Nanjing University Animal Care and Use Committee.

## Supporting information


Data S1.

## Data Availability

Data Availability Statement All data presented or analysed in present study are included in this manuscript.
