# Supplementary material for: ADT‐OH synergistically enhanced the antitumor activity of celecoxib in human colorectal cancer cells
Source: Cancer Med. 2023 Jul 26;12(16):17193–211. doi: 10.1002/cam4.6342 (PMC10501245; doi:10.1002/cam4.6342)
Supplement: Supplementary file 1 — Data S1. [file CAM4-12-17193-s001.docx]

Supplementary Material

**Supplementary Tables and Figures**

**Table S1: The primers of selected genes for real-time PCR**

| **Gene** | **Forward primer (5’-3’)** | **Reverse primer (5’-3’)** |
| --- | --- | --- |
| **β-actin** | GAGACCTTCAACACCCCAGC | ATGTCACGCACGATTTCCC |
| **MMP-2** | CTTCTTCAAGGACCGGTTCA | GCTGGCTGAGTACCAGTA |
| **MMP-9** | TGGGCTACGTGACCTATGAC | GCCCAGCCCACCTCCACTCC |
| **Cofilin** | TCCTTGACCTCCTCGTAGCA | TGCGCCCCTTAAGAGCAAA |
| **LIMK** | ACTGCGGGCACTGCTACTA | GCTTGCCATGAGATGAGGCT |
| **ROCK** | TCTGAAAGGAGGGACCGAACC | GTTCCTGTTTGTGTCGAGCCATCA |

**Table S2: The IC_50_ value of ADT-OH and Celecoxib after the treatment of NCM460, HCT116 and CT26 for 48 h**

| **Drug treatment** | **ADT-OH** | **Celecoxib** |
| --- | --- | --- |
| **IC_50_ value (μM) for NCM460** | 115.59 | 79.63 |
| **IC_50_ value (μM) for HCT116** | 63.63 | 52.05 |
| **IC_50_ value (μM) for CT26** | 67.81 | 56.21 |

**Figure S1: ADT-OH combined with celecoxib causes cell cycle arrest at the G0/G1 phase in CT26 cells**

**
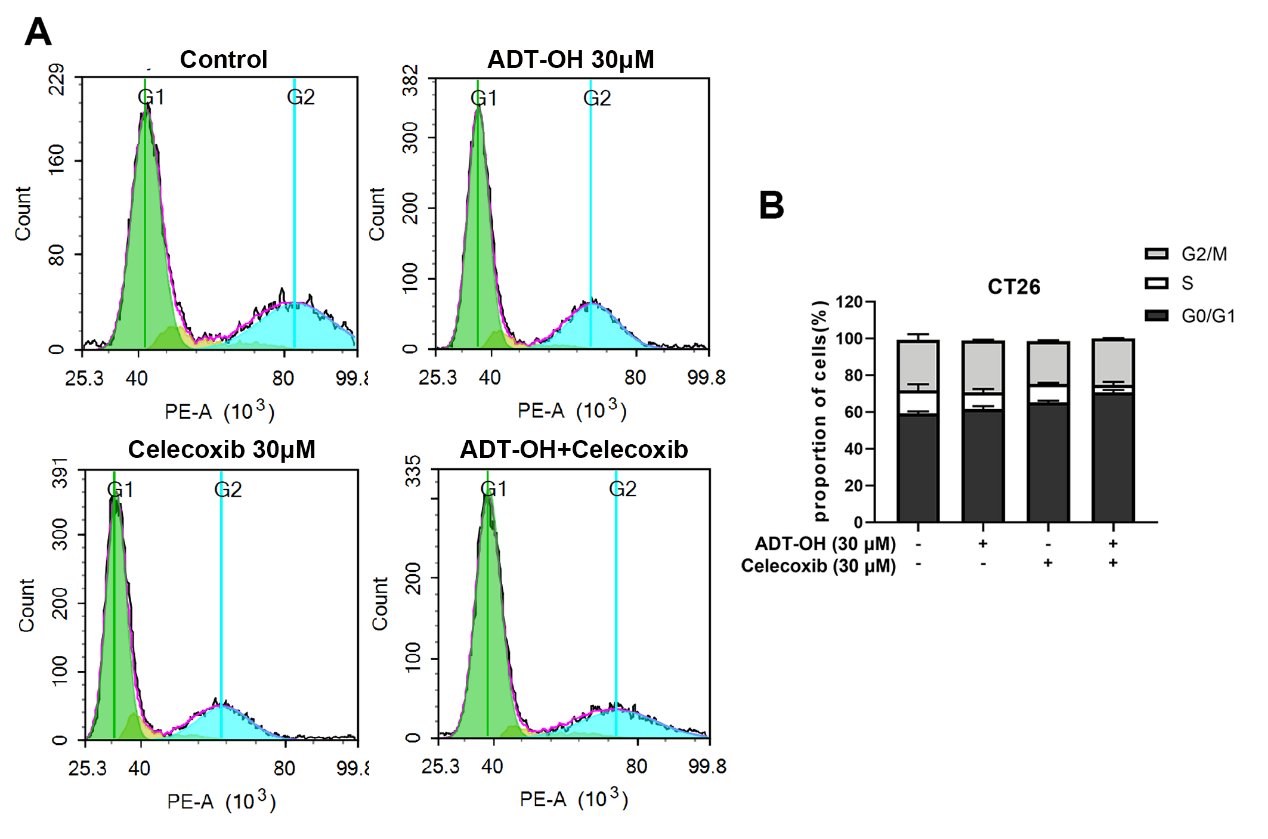
**

**Figure S1: ADT-OH combined with celecoxib causes cell cycle arrest at the G0/G1 phase in CT26 cells.** (**A**) The cell cycle distribution of CT26 cells was detected by flow cytometry after 48 h of treatment with ADT-OH (30 μM) and/or celecoxib (30 μM). (**B**) Statistics of cell cycle distribution. Data are presented as mean ± SD.

**Figure S2: Cofilin and LIMK are overexpressed in a variety of cancers including colorectal cancer**

**
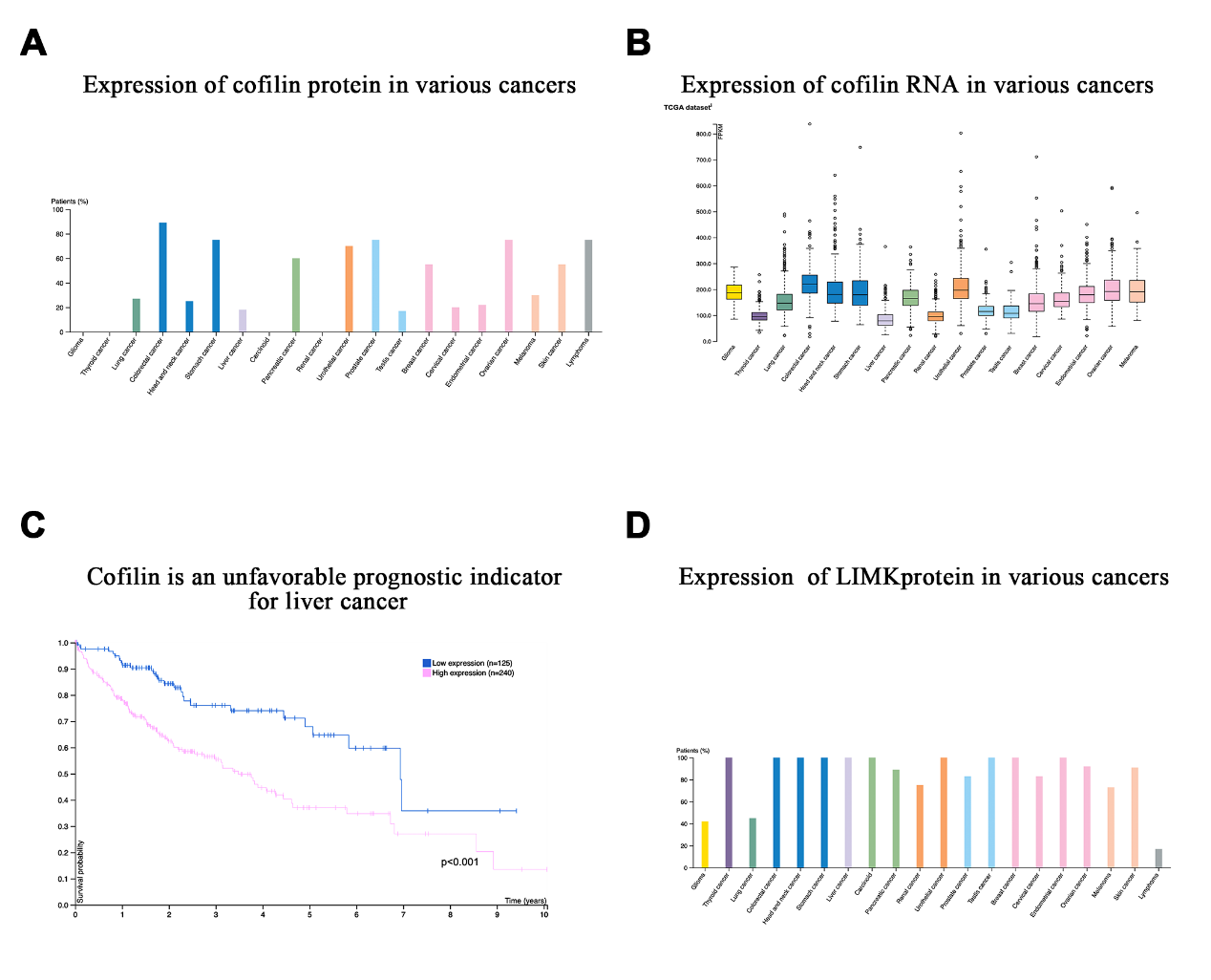
**

**Figure S2: Cofilin and LIMK are overexpressed in a variety of cancers including colorectal cancer.** (**A**) The protein expression level of cofilin in various cancers. (**B**) The RNA expression level of cofilin in various cancers. (**C**) Cofilin is an unfavorable prognostic indicator for liver cancer (**D**) The protein expression level of LIMK in various cancers. Data were obtained from the Human Protein Atlas database. (URL: https://www.proteinatlas.org/).

**Figure S3: ADT-OH combined with celecoxib promotes ROS production in CT26 cells**


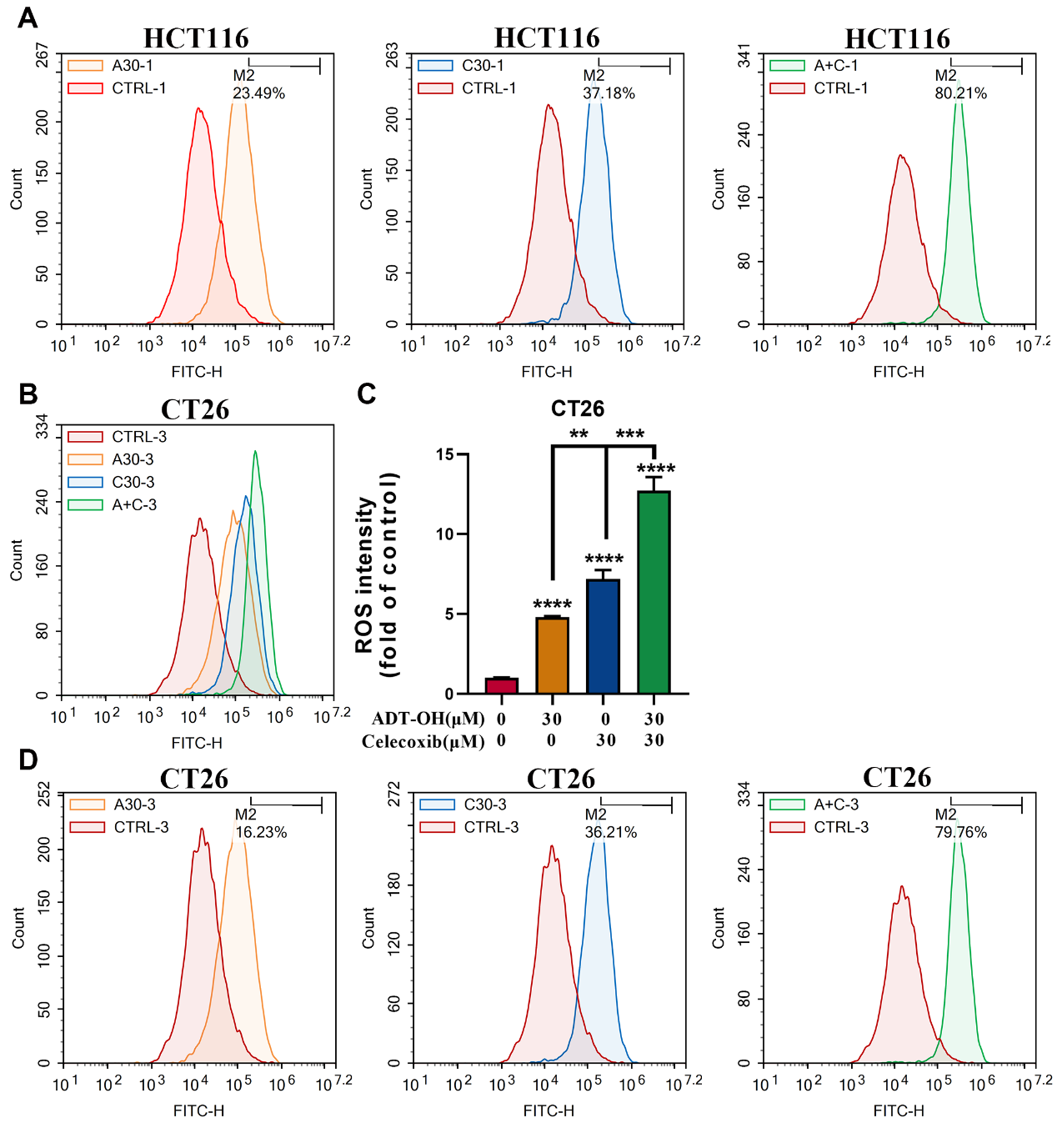


**Figure S3: ADT-OH combined with celecoxib promotes ROS production in CT26 cells.** (**A**) HCT116 cells were treated with ADT-OH (30 µM) and/or celecoxib (30 µM) for 48 h, and flow cytometry was used to detect the level of ROS. (**B**) CT26 cells were treated with ADT-OH (30 µM) and/or celecoxib (30 µM) for 48 h, and flow cytometry was used to detect the level of ROS. (**C**) Statistics of ROS levels in CT26 cells with different drug treatments. (**D**) CT26 cells were treated with ADT-OH (30 µM) and/or celecoxib (30 µM) for 48 h, and flow cytometry was used to detect the level of ROS. Data are presented as mean ± SD. ***p < 0.01, ***p < 0.001, ****p < 0.0001*.

**Figure S4: ADT-OH combined with celecoxib promotes CT26 cell apoptosis**


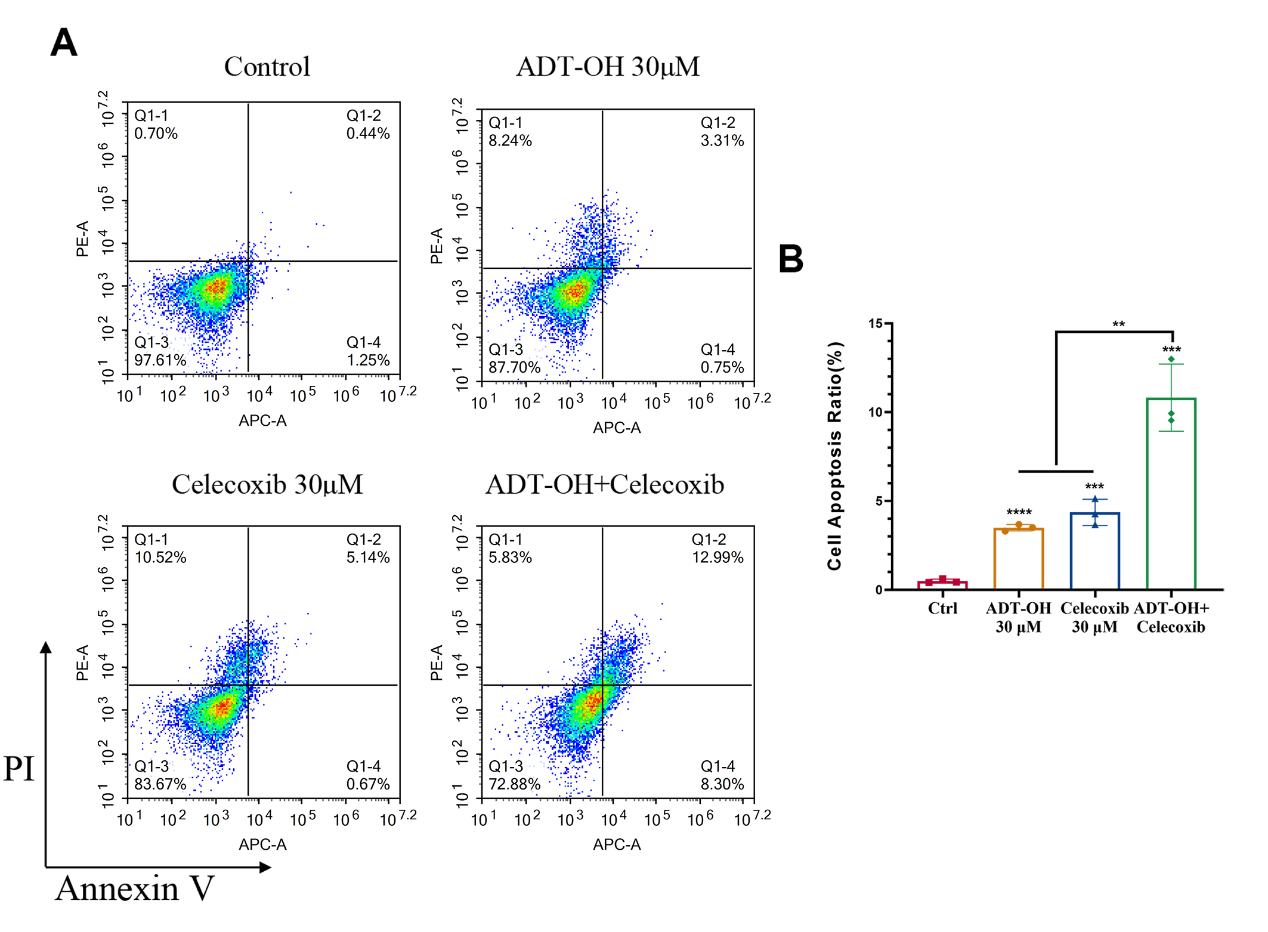


**Figure S4: ADT-OH combined with celecoxib promotes CT26 cell apoptosis.** (**A**) After 48 h of treatment with ADT-OH (30 µM) and/or celecoxib (30 µM), the level of apoptosis of CT26 cells was detected by flow cytometry. (**B**) Statistics of apoptosis levels in CT26 cells with different drug treatments. Data are presented as mean ± SD. ***p < 0.01*, ****p < 0.001*, *****p < 0.0001*.

**Figure S5: ADT-OH combined with celecoxib had no obvious toxic and side effects in nude mice at the dose we used**


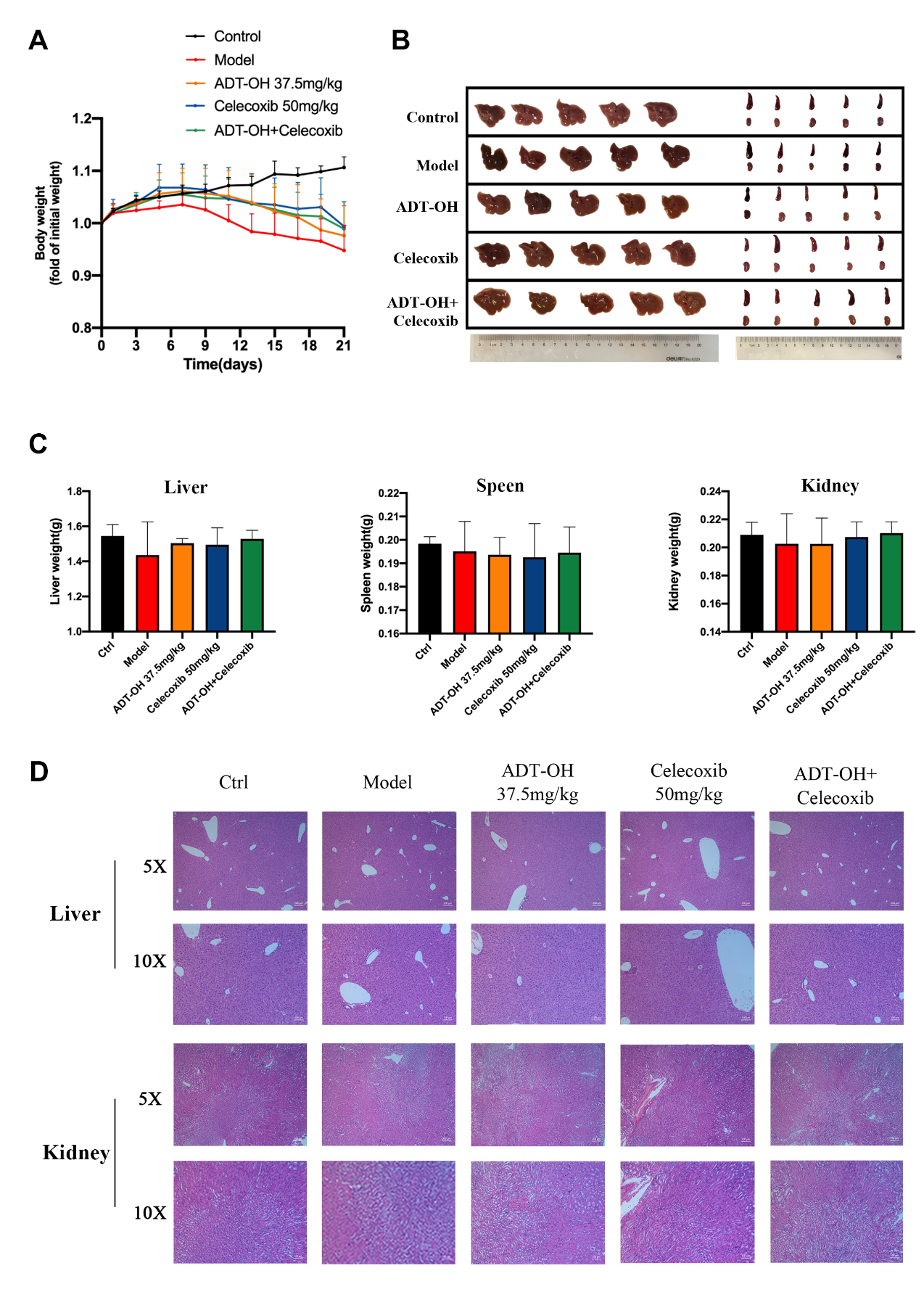


**Figure S5: ADT-OH combined with celecoxib had no obvious toxic and side effects in nude mice at the dose we used.** (**A**) Continuous monitoring of nude mice's body weight during treatment. (**B**) After the treatment, the liver, spleen and kidney of nude mice were dissected. (**C**) Statistics of liver, spleen and kidney weights in nude mice of each group. (**D**) H&E staining of nude mouse liver and kidney. Data are represented as mean ± SD. n = 6 mice in each group.
